# A nomogram model to predict death rate among non-small cell lung cancer (NSCLC) patients with surgery in surveillance, epidemiology, and end results (SEER) database

**DOI:** 10.1186/s12885-020-07147-y

**Published:** 2020-07-17

**Authors:** Bo Jia, Qiwen Zheng, Jingjing Wang, Hongyan Sun, Jun Zhao, Meina Wu, Tongtong An, Yuyan Wang, Minglei Zhuo, Jianjie Li, Xue Yang, Jia Zhong, Hanxiao Chen, Yujia Chi, Xiaoyu Zhai, Ziping Wang

**Affiliations:** 1 Key Laboratory of Carcinogenesis and Translational Research (Ministry of Education/Beijing), Department of Thoracic Medical Oncology, Peking University Cancer Hospital & Institute, 52 Fucheng Road, Haidian District, Beijing, 100142 China; 2 Department of Epidemiology and Biostatistics, School of Public Health, Peking University, Beijing, China; 3 Department of General Practice, The Third Affiliated Hospital, Sun Yat_Sen University, Guangzhou, China

**Keywords:** NSCLC, Surgery, Prognosis, SEER, Nomogram

## Abstract

**Background:**

This study aimed to establish a novel nomogram prognostic model to predict death probability for non-small cell lung cancer (NSCLC) patients who received surgery..

**Methods:**

We collected data from the Surveillance, Epidemiology, and End Results (SEER) database of the National Cancer Institute in the United States. A nomogram prognostic model was constructed to predict mortality of NSCLC patients who received surgery.

**Results:**

A total of 44,880 NSCLC patients who received surgery from 2004 to 2014 were included in this study. Gender, ethnicity, tumor anatomic sites, histologic subtype, tumor differentiation, clinical stage, tumor size, tumor extent, lymph node stage, examined lymph node, positive lymph node, type of surgery showed significant associations with lung cancer related death rate (*P* < 0.001). Patients who received chemotherapy and radiotherapy had significant higher lung cancer related death rate but were associated with significant lower non-cancer related mortality (P<0.001). A nomogram model was established based on multivariate models of training data set. In the validation cohort, the unadjusted C-index was 0.73 (95% CI, 0.72–0.74), 0.71 (95% CI, 0.66–0.75) and 0.69 (95% CI, 0.68–0.70) for lung cancer related death, other cancer related death and non-cancer related death.

**Conclusions:**

A prognostic nomogram model was constructed to give information about the risk of death for NSCLC patients who received surgery.

## Background

The morbidity and mortality of lung cancer ranked the first in China and globally [1, 2]. Non-small cell lung cancer (NSCLC) accounts for about 75 to 80% of lung cancer patients, thus the treatment of NSCLC has been an urgent health issue worldwide.

Radical surgery is required for early stage and parts of locally advanced NSCLC patients [3]. Survival of NSCLC patients after surgery varies greatly, and previous reported prognostic factors include age, tumor size, metastatic lymph node numbers, clinical stage, etc. [4–6] However, other factors such as ethnicity, surgical method, primary tumor location, anatomic sites, histological subtype, etc. remain controversial. Therefore, studies with larger sample data and more rigorous statistical method assessing this problem are still needed.

For the reason that some early stage NSCLC patients who received radical surgery may have relative long-term survival, several other causes of death may occur among NSCLC patients. But previous studies mainly focus on investigating prognostic factors for lung cancer related death, studies considering non-cancer related death are inadequate.

To better evaluate the prognosis of resected NSCLC patients, and therefore to further provide more optimal treatment strategies for these patients, we estimated the causes of lung cancer related, other cancer related, and non cancer related death among patients in a population based Surveillance, Epidemiology, and End Results (SEER) cohort using a innovative and validated nomogram model.

## Methods

### Data source

We collected data from the SEER database of National Cancer Institute in the United States [7]. The data was obtained using the SEER* Stat. The North American Association of Central Cancer Registries (NAACCR) documented data items and codes [8]. Primary cancer histology and site were coded by the 3rd edition of the International Classification of Diseases for Oncology (ICD-O-3).

### Cohort selection

**Fig. 1:**
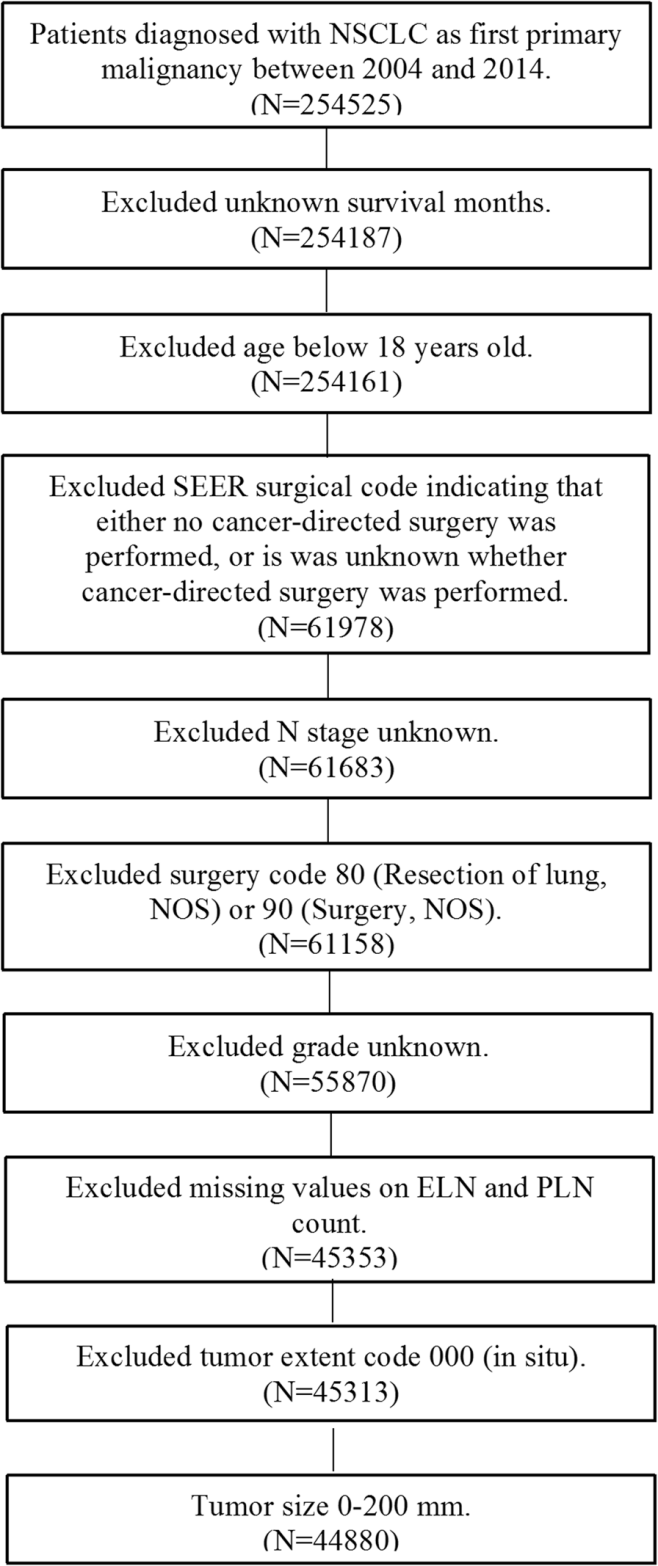
Flow chart of patients’ selection

Patients with lung tumors (site codes, C34.0-C34.9) were included in this study from the year 2004 to 2014. The following histologic codes were designated as NSCLC: 8010, 8012, 8013, 8014,8015, 8020,8021,8022,8031,8032, 8046, 8050–8052, 8070–8078, 8140–8147, 8250–8255, 8260, 8310,8323, 8430, 8480, 8481,8482, 8490, 8560, and 8570–8575. Patients who did not receive radical surgery or aged 18 years or younger were excluded. In accordance with the requirement of using SEER database [9], we obtained the data agreement. Figure 1 displayed the flow chart of patients’ selection procedure in this study. SEER database conducted the follow-up for all patients, and the information of patients’ follow-up time, survival status and survival time were all recorded. Therefore we could investigate the follow-up time and OS for these patients. In this study, the missing data that could not use to assess the survival status was eliminated before statistics.

### Statistical analysis

Demographic and clinical variables adopted in the further analysis included age, gender, ethnicity, primary tumor location, anatomic sites, histological subtype, tumor extent, differentiation, clinical stage, tumor size, lymph node involvement, examined lymph node (ELNs), positive lymph node (PLNs), chemotherapy and radiotherapy. Categorical variables were grouped for clinical reasons, and the decisions regarding grouping were made before data analysis. Mean, medians and ranges were reported for continuous variables, as appropriate. Frequencies and proportions were reported for categorical variables.

The primary endpoint of this study was cause-specific survival. According to the COD code, we defined the cause of death into three groups: lung cancer related, other cancer related and non-cancer related. Cumulative incidence function (CIF) was used to illustrate death rate. The CIF was compared across groups by using Gray’s test [10]. Fine and Gray competing risks proportional hazards regressions was performed to predict five- and ten-year probabilities of the three causes of death [11]. For nomogram construction, two thirds of the patients were randomly assigned to the training data set (*n* = 31,415) and one third to the validation data set (*n* = 13,465). We used restricted cubic splines with three knots at the 10, 50, and 90% empirical quantiles to model continuous variables [12]. A model selection technique based on the Bayesian information criteria was employed to avoid overfitting when establishing competing risk models (eTable S1) [13].

The performance of the nomogram included its discrimination and calibration was tested using the validation data set. Discrimination is the ability of a model to separate subject outcomes, which is indicated by Harrell C index [14, 15]. Calibration, which compares predicted with actual survival, was evaluated with a calibration plot. We used the validation set to compare the final reduced model-predicted probability of death with the observed 5 and 10-year cumulative incidence of death. The predictions were supposed to fall on a 45-degree diagonal line if the model was well calibrated. In addition, the bootstrapping technique was used for internal validation of the developed model based on 1000 resamples.

The R software (version 3.3.3; http:// www.r-project.org) was performed for all statisitcal analysis. We used R packages cmprsk, rms and mstate for modeling and developing the nomogram. The reported significance levels were all two-sided, with statistical significance set at 0.05.

## Results

### Patient characteristics

A total of 44,880 NSCLC patients who received surgery from 2004 to 2014 were included in this study. Most patients were diagnosed at stage I (62%), were Caucasians (83.5%) and received lobectomy (82.9%). The median diagnostic age was 67 years. The median follow-up time was 31 months (IQR 12 to 61 months), and for still alive patients, the median follow-up time was 42 months (IQR 17–74 months). At last follow up, the death rate was 41.9%, with 12,958 patients (28.9%) died from lung cancer, 510 (1.1%) died from other cancers, and 5357 (11.9%) died from non-cancer causes. The most frequent other cancer death were resulted from miscellaneous malignant cancer (54.5%), brain and other nervous system (6.9%) and pancreas (3.5%) cancers. The most frequent non-cancer deaths were resulted from diseases of heart (28.3%), chronic obstructive pulmonary disease and associated conditions (19.8%) and cerebrovascular diseases (5.8%) (Table 1).

**Table 1:** Patient Characteristics

Characteristics	All Patient		Training Cohort		Validation Cohort	
	(*N* = 44,880)		(*N* = 31,415)		(*N* = 13,465)	
	Number	%	Number	%	Number.	%
Diagnostic Age, years						
Mean	66.7		66.7		66.8	
Median	67		67		67	
Range	18–101		18–101		18–96	
Gender						
Female	22,737	50.7	15,884	50.6	6853	50.9
Male	22,143	49.3	15,531	49.4	6612	49.1
Ethnicity						
White	37,487	83.5	26,316	83.8	11,171	83
Asian	3159	7	2160	6.9	999	7.4
Black	3939	8.8	2742	8.7	1197	8.9
Others/Unknown	295	0.7	197	0.6	98	0.7
Primary tumor location						
Left-sided	18,752	41.8	13,103	41.7	5649	42
Right-sided	26,128	58.2	18,312	58.3	7816	58
Anatomic sites						
Upper	26,831	59.8	18,766	59.7	8065	59.9
Middle	2152	4.8	1491	4.7	661	4.9
Lower	14,237	31.7	9940	31.6	4297	31.9
Bronchus/Others	1660	3.7	1218	3.9	442	3.3
Histologic subtype						
ADC	21,933	48.9	15,321	48.8	6612	49.1
SCC	12,593	28.1	8871	28.2	3722	27.6
BAC	4746	10.6	3292	10.5	1454	10.8
ADSC	1279	2.8	909	2.9	370	2.7
LCC	1279	2.8	900	2.9	379	2.8
Others	1327	3	923	2.9	404	3
Unspecified	1723	3.8	1199	3.8	524	3.9
Differentiation						
Well	6146	13.7	4292	13.7	1854	13.8
Moderately	19,882	44.3	13,884	44.2	5998	44.5
Poorly	17,783	39.6	12,485	39.7	5298	39.3
Undifferentiated	1069	2.4	754	2.4	315	2.3
Clinical stage						
I	27,825	62	19,476	62	8349	62
II	6715	15	4681	14.9	2034	15.1
III	7982	17.8	5653	18	2329	17.3
IV	2358	5.3	1605	5.1	753	5.6
Tumor size, cm						
Mean	3.4		3.4		3.4	
Median	2.8		2.8		2.8	
Range	1–20		1–20		1–20	
Tumor extent						
Local	29,526	65.8	20,649	65.7	8877	65.9
Regional	14,836	33.1	10,404	33.1	4432	32.9
Distant	518	1.2	362	1.2	156	1.2
Lymph node stage						
N0	32,207	71.8	22,539	71.7	9668	71.8
N1	6809	15.2	4733	15.1	2076	15.4
N2	5700	12.7	4027	12.8	1673	12.4
N3	164	0.4	116	0.4	48	0.4
Examined lymph node						
Mean	9.9		10		9.9	
Median	8		8		8	
Range	1–90		1–90		1–90	
Positive lymph node						
Mean	0.8		0.8		0.8	
Median	0		0		0	
Range	0–41		0–41		0–39	
Type of surgery						
Lobectomy	37,203	82.9	26,056	82.9	11,147	82.8
Pneumonectomy	2830	6.3	1978	6.3	852	6.3
Sub-lobar	4847	10.8	3381	10.8	1466	10.9
Chemotherapy						
None	31,835	70.9	22,214	70.7	9621	71.5
Yes	13,045	29.1	9201	29.3	3844	28.5
Radiotherapy						
None	39,049	87	27,357	87.1	11,692	86.8
Yes	5831	13	4058	12.9	1773	13.2
Lung cancer related death	12,958	28.9	9154	29.1	3804	28.3
Other cancer related death	510	1.1	352	1.1	158	1.2
Non-cancer related death	5357	11.9	3743	11.9	1614	12
Follow-up, months						
Mean	39.8		39.8		39.9	
Median	31		30		31	
Range	0–131		0–131		0–131	

*ADC* adenocarcinoma, *ASDC* adenosquamous carcinoma, *BAC* bronchoalveolar carcinoma, *SCC* squamous cell carcinoma, *LCC* large cell carcinoma

### Survival

Lung cancer related, other cancer related and non-cancer related death probability were shown in eFigure S1, S2, S3 and S4. Diagnostic age, gender, ethnicity, anatomic sites, histologic subtype, differentiation status, clinical stage, tumor size, tumor extent, examined lymph node, surgery type, showed significant relationships with overall survival (*P*<0.001) (eTable S2). Five- and 10-year lung cancer related death probability increased with age, stage, tumor size, tumor extent, lymph node stage, positive lymph node numbers (*P*<0.001). Male patients had higher lung cancer-related death rate compared with female patients (*P*<0.001). Ethnicity, histologic subtype, anatomic sites of lung cancer, examined lymph node, differentiation status, surgery type, showed significant relationships with lung cancer related death probability (*P*< 0.001). Patients who received chemotherapy and radiotherapy had significant higher lung cancer related mortality for NSCLC patients with surgery but were associated with significant lower non-cancer related death rates (*P*<0.001) **(**Table 2**)**.

**Table 2:** Five and 10-year lung cancer related, other cancer related and non-cancer related death probability

Characteristics	Lung cancer related death probability			Other cancer related death probability			Non-cancer related death probability		
	5 Year	10 Year	P	5 Year	10 Year	P	5 Year	10 Year	P
	(%)	(%)		(%)	(%)		(%)	(%)	
Diagnostic Age, years			< 0.001			0.159			< 0.001
< 45	28.1	36.9		0.4	0.4		4.5	8.2	
45–64	31.7	39.6		1.4	1.6		7.3	14.2	
65–74	33.6	41.4		1.2	1.9		12.3	23.3	
≥ 75	37.0	44.3		1.4	1.7		19.6	34.2	
Gender			< 0.001			0.146			< 0.001
Female	29.9	38.7		1.2	1.7		9.8	19.3	
Male	37.3	44.1		1.4	1.7		14.2	24.9	
Ethnicity			< 0.001			< 0.001			< 0.001
White	33.8	41.5		1.3	1.7		12.4	22.7	
Asian	31.2	41.4		0.8	1.2		8.5	16.1	
Black	34.2	40.8		2.0	2.2		10.8	20.4	
Others/Unknown	23.7	24.8		0.3	0.3		9.8	36.1	
Primary tumor location			0.09			0.676			0.097
Left-sided	34.1	41.9		1.3	1.7		12.2	23.2	
Right-sided	33.3	41.0		1.3	1.7		11.9	21.4	
Anatomic sites			< 0.001			0.45			0.032
Upper	31.9	39.2		1.3	1.7		12.0	23.1	
Middle	33.6	41.5		1.2	1.5		11.7	18.9	
Lower	35.2	44.0		1.2	1.7		12.4	21.4	
Bronchus/Others	47.3	53.2		1.7	1.9		10.8	16.6	
Histologic subtype			< 0.001			0.04			< 0.001
ADC	33.4	42.2		1.3	1.7		10.3	19.6	
SCC	35.2	40.9		1.3	1.6		16.6	29.1	
BAC	23.8	33.8		0.8	1.5		8.4	16.2	
ADSC	41.7	48.7		1.6	1.7		12.7	21.8	
LCC	43.7	49.8		2.1	2.3		13.1	20.6	
Other	29.0	40.6		1.1	1.1		7.2	17.9	
Unspecified	41.4	45.6		1.9	2.2		11.3	20.1	
Differentiation			< 0.001			< 0.001			< 0.001
Well	17.3	26.5		0.7	1.2		9.1	20.5	
Moderately	31.5	40.2		1.1	1.6		12.8	22.4	
Poorly	40.7	47.0		1.6	1.9		12.1	22.1	
Undifferentiated	41.3	47.8		1.9	2.1		12.8	21.2	
Clinical stage			< 0.001			< 0.001			< 0.001
I	22.0	30.0		1.0	1.4		13.1	25.7	
II	46.5	53.1		1.5	1.9		11.8	18.7	
III	53.5	61.1		1.8	2.2		9.7	16.1	
IV	62.8	71.3		2.6	2.6		8.4	11.9	
Tumor size, cm			< 0.001			< 0.001			< 0.001
≤ 1.0	18.4	27.4		0.8	1.9		8.8	18.3	
1.1 to 3.0	26.2	34.5		1.1	1.6		12.3	23.8	
3.1 to 5.0	39.6	47.2		1.5	1.7		12.8	22.6	
5.1 to 7.0	47.6	53.8		1.5	1.9		11.0	17.9	
> 7.1	57.6	62.1		1.8	2.5		10.2	15.6	
Tumor extent			< 0.001			< 0.001			< 0.001
Local	28.0	35.9		1.1	1.5		12.5	23.8	
Regional	60.3	65.9		2.0	2.7		13.1	16.9	
Distant	43.8	51.3		1.6	2.0		11.0	19.1	
Lymph node stage			< 0.001			< 0.001			< 0.001
N0	25.2	33.2		1.1	1.5		12.9	25.0	
N1	49.6	56.7		1.5	2.0		10.9	16.6	
N2/N3	59.0	66.3		1.9	2.2		8.8	14.0	
Examined lymph node			< 0.001			0.379			< 0.001
< 5	34.5	42.7		1.5	1.9		13.2	24.7	
5 to 9	32.5	40.1		1.1	1.6		12.0	22.0	
10 to 14	32.8	40.2		1.2	1.6		11.5	21.8	
15 to 20	34.0	42.1		1.3	1.5		10.5	18.7	
≥ 20	36.2	43.0		1.4	1.7		11.4	18.3	
Positive lymph node			< 0.001			< 0.001			< 0.001
0	25.7	33.5		1.1	1.5		12.8	24.9	
1	49.6	56.8		1.9	2.4		10.5	17.0	
2	52.3	59.9		1.6	1.7		10.2	15.3	
3	55.6	63.7		1.4	2.0		10.4	14.9	
≥ 4	63.7	70.9		1.8	1.9		8.8	11.4	
Type of surgery			< 0.001			0.249			< 0.001
Lobectomy	32.0	39.7		1.2	1.7		11.8	22.0	
Pneumonectomy	51.0	57.6		1.7	1.8		11.5	17.4	
Sub-lobar	35.7	43.9		1.3	1.8		14.4	26.7	
Chemotherapy			< 0.001			0.214			< 0.001
None	28.2	35.5		1.2	1.7		14.0	26.1	
Yes	46.4	54.9		1.4	1.8		7.3	13.0	
Radiotherapy			< 0.001			< 0.001			< 0.001
None	30.0	37.7		1.2	1.6		12.5	23.3	
Yes	56.9	64.3		1.9	2.1		8.8	14.8	

### Nomogram prognositc model

**Fig. 2:**
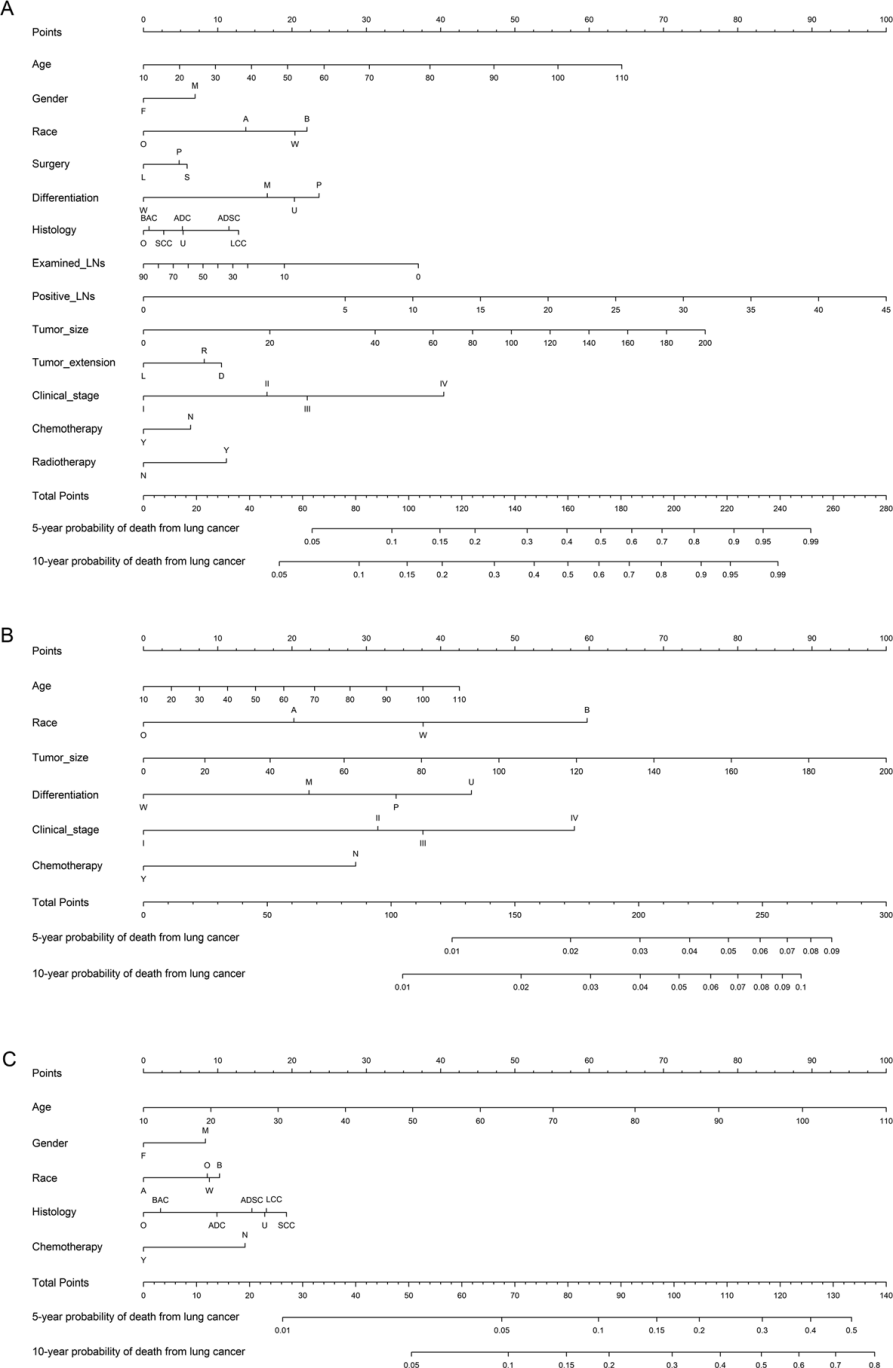
Nomogram model to predict 5- and 10-year (**a**) lung cancer, related (**b**) other cancer related, and (**c**) non-cancer related death rate in resected NSCLC patients. Gender: F, female; M, male; Ethnicity: B, black; O, other; W, white; A, asian; Surgery: L, lobectomy; P, pneumonectomy; S, sub-lobar; Differentiation: W, well differentiated; M, moderately differentiated; P, poorly differentiated; U, undifferentiated; Histology: ADC, adenocarcinoma; ASDC, adenosquamous carcinoma; BAC, bronchoalveolar carcinoma; SCC, squamous cell carcinoma; LCC, large cell carcinoma; O, other; U, unspecified NSCLC; Tumor extension: D, distant; L, localized; R, regional; Chemotherapy: N, none; Y, received chemotherapy; Radiotherapy: N, none; Y, received radiotherapy

**Fig. 3:**
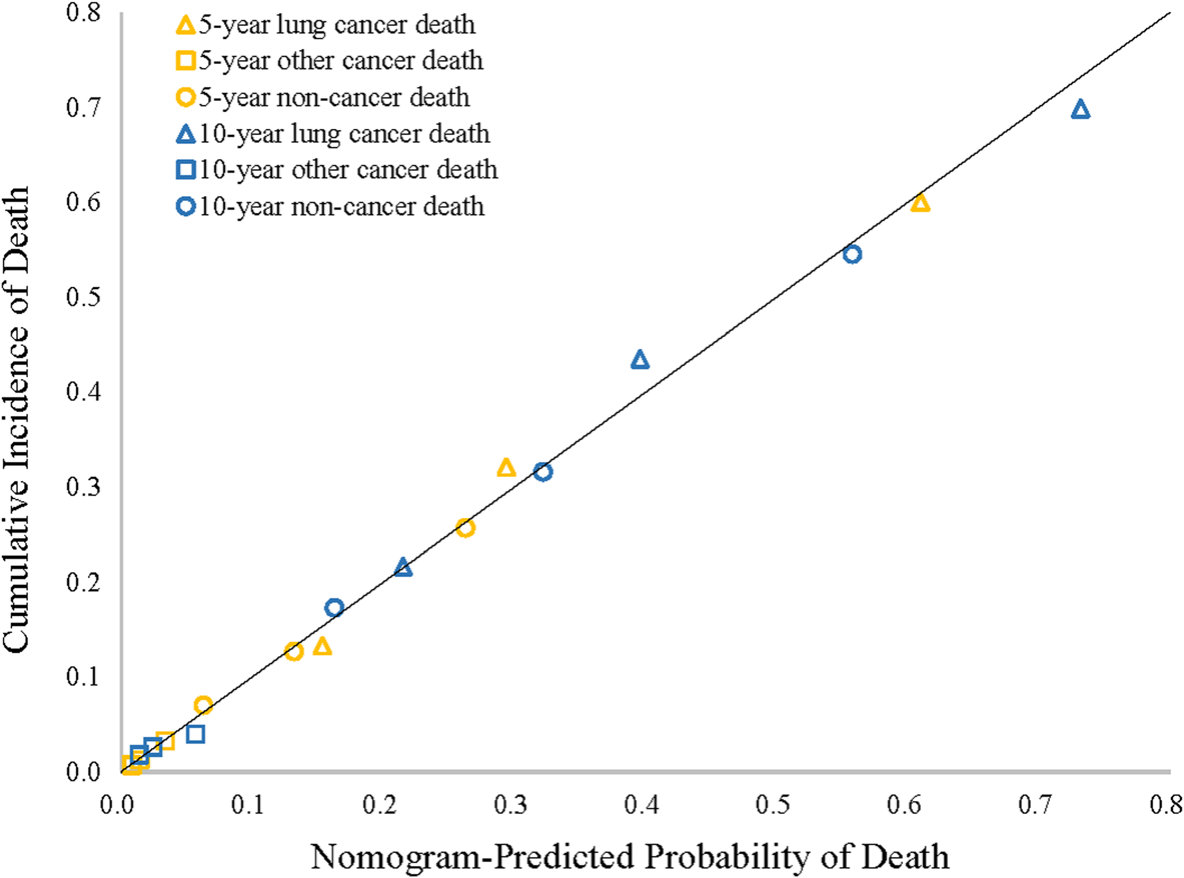
Nomogram calibration plot in the validation set. The x-axis represents the mean predicted death probability. The y-axis represents actual death rate. The solid line represents equality between the predicted and actual probability

A nomogram model was established based on multivariate models of training data set. We could calculate the 5- or 10-year death rate by this nomogram prognositic model **(**Fig. 2). Schoenfeld−type residuals of a proportional sub distribution hazard model for lung cancer related deaths were shown in eFigure S5. In the validation cohort, the unadjusted C-index was 0.73 (95% CI, 0.72–0.74), 0.71 (95% CI, 0.66–0.75) and 0.69 (95% CI, 0.68–0.70) for lung cancer related death, other cancer related death and non-cancer related death. This indicated that the models are convincingly precise. Figure 3 illustrated the CIF plot calibration. Good coincidence between predicted and actual outcomes was observed because the points are close to the 45-degree line.

## Discussion

To our knowledge, this is the largest population based study establishing a novel nomogram prognostic model predicting lung cancer related death rate, other cancer related death rate, and non–cancer related death rate for NSCLC patients who received surgery in SEER database.

Recent studies showed that several factors including tumor size, lymph node metastasis, clinical stage, age, etc. were associated with long time survival for lung cancer patients with surgery. However, the results were heterogeneous for the reason that most studies evaluating the prognosis of NSCLC had relative short follow-up with limited sample size. Therefore larger sample data with more validated and rigorous statistical methods were required. Besides, the population-based SEER database could be used with the ability to assess this issue on a larger sample with long follow-up, which can effectively avoid biases. In this study, was collected a large population of 44,880 resected NSCLC patients in SEER database.

Moreover, to make the bias minimized, we used a novel and validated prognostic model. Nomogram has been considered as a trustworthy method to generate more accurate prediction of prognosis [16–18]. The performance of the nomogram may also have discrimination, thus calibration should be conducted using a validation data set. Our study showed, the unadjusted C-index was 0.73 (95% CI, 0.72–0.74), 0.71 (95% CI, 0.66–0.75) and 0.69 (95% CI, 0.68–0.70) for lung cancer related death, other cancer related death and non-cancer related death in the validation cohort. This indicated that the models are convincingly precise. Besides, our study showed good coincidence between predicted and actual outcomes because the points are close to the 45-degree line.

Our study showed 5- and 10-year lung cancer related death probability increased with age, stage, tumor size, tumor extent, lymph node involvement, positive lymph node numbers which were consistent with previous studies [3–6]. In our study, male patients had higher lung cancer-related death rate compared with female patients. Several studies have demonstrated that epidermal growth factor receptor (EGFR) - tyrosine kinase inhibitors (TKIs) could noticeably improve survival of EGFR positive mutation advanced NSCLC patients [19–22]. EGFR mutation is the most common gene mutation in Asian female lung adenocarcinoma patients, therefore the prognosis of female lung cancer patients might be better. Our study showed patients with radiotherapy were associated with a significantly higher lung cancer related death rate. Radiotherapy was always performed to patients with more aggressive stage or, mediastinal lymph node metastasis and these patients may originally have poor prognosis. However, the appropriate opportunity and indication of radiotherapy still need further investment.

Previous studies mainly focus on investigating lung cancer related survival for NSCLC patients, studies with concern of other causes of death are limited. In SEER database, the data of survival status, survival months, cause-specific death classification was available and death resulting from other cancer and non-cancer was also recorded. Therefore we could investigate calculate lung cancer related, other cancer related and non-cancer related death probability using these data. We divided cause of death into lung cancer related, other cancer related and non-cancer related. In our study, the most frequent non-cancer deaths were resulted from diseases of heart, chronic obstructive pulmonary disease and associated conditions, and cerebrovascular diseases. Therefore the complications of heart and respiratory system during treatment procedures require closer monitoring.

There were also some limitations in this study. First, some variables are not recorded in SEER database, such as disease progression time, specific chemotherapy regimens, etc. Besides, we did not use the 7th or 8th AJCC staging system in this study. We selected patients in the SEER database from 2004 to 2014. The 6th AJCC staging system was applied for all patients during the decade. But the 7th AJCC staging system had not been widely used before 2010. The 8th AJCC staging system was applied after 2017. Stage information from 2004 to 2010 could not be accessed when using the 7th or 8th AJCC staging system. For the huge sample size, re-classification of patients was impossible. But there was no significant difference between stage I to stage III patients according to different staging systems, which had no significant impact on the study results.

## Conclusions

A novel prognostic nomogram model using a large population based database was constructed to predict mortality for NSCLC patients who received surgery. This validated prognostic model may be helpful to give information about the risk of death for these patients.

## Supplementary information

**Additional file 1: eTable S1.** Proportional Subdistribution Hazards Models of Death Rate. **eTable S2.** Prognostic factors for overall survival by multivariable Cox regression. **eFigure S1.** Lung cancer related, other cancer related and non-cancer related death rates by (A) age, (B) gender, (C) race and (D) primary tumor location. **eFigure S2.** Lung cancer related, other cancer related and non-cancer related death rates by (E) Anatomic sites, (F) histology subtype, (G) differentiation and (H) clinical stage. **eFigure S3.** Lung cancer related, other cancer related and non-cancer related death rates by (I) tumor size, (J) tumor extent, (K) lymph node involvement and (L) examined lymph nodes. **eFigure S4.** Lung cancer related, other cancer related and non-cancer related death rates by (M) positive lymph nodes, (N) surgery, (O) chemotherapy and (P) radiotherapy. **eFigure S5.** Schoenfeld−type residuals of a proportional subdistribution hazard model for lung cancer related deaths.

## Data Availability

Data files were downloaded directly from the SEER website.

## Abbreviations

ADC: Adenocarcinoma
ASDC: Adenosquamous carcinoma
BAC: Bronchoalveolar carcinoma
HR: Hazard ratio
ICD-O: International Classification of Diseases for Oncology
LCC: Large cell carcinoma
NAACCR: North American Association of Central Cancer Registries
NSCLC: Non-small cell lung cancer
OS: Overall survival
SEER: Surveillance, Epidemiology, and End Results
SCC: Squamous cell carcinoma
