# Supplementary material for: A nomogram model to predict death rate among non-small cell lung cancer (NSCLC) patients with surgery in surveillance, epidemiology, and end results (SEER) database
Source: BMC Cancer. 2020 Jul 17;20:666. doi: 10.1186/s12885-020-07147-y (PMC7367407; doi:10.1186/s12885-020-07147-y)
Supplement: Supplementary file 1 — Additional file 1: eTable S1. Proportional Subdistribution Hazards Models of Death Rate. eTable S2. Prognostic factors for overall survival by multivariable Cox regression. eFigure S1. Lung cancer related, other cancer related and non-cancer related death rates by (A) age, (B) gender, (C) race and (D) primary tumor location. eFigure S2. Lung cancer related, other cancer related and non-cancer related death rates by (E) Anatomic sites, (F) histology subtype, (G) differentiation and (H) clinical stage. eFigure S3. Lung cancer related, other cancer related and non-cancer related death rates by (I) tumor size, (J) tumor extent, (K) lymph node involvement and (L) examined lymph nodes. eFigure S4. Lung cancer related, other cancer related and non-cancer related death rates by (M) positive lymph nodes, (N) surgery, (O) chemotherapy and (P) radiotherapy. eFigure S5. Schoenfeld−type residuals of a proportional subdistribution hazard model for lung cancer related deaths. [file 12885_2020_7147_MOESM1_ESM.docx]

eTable 1. Proportional Subdistribution Hazards Models of Death Rate

| Characteristics | Lung cancer related | | | | Other cancer related | | | | Non-cancer related | | | |
| --- | --- | --- | --- | --- | --- | --- | --- | --- | --- | --- | --- | --- |
|  | Full Model | | Reduced Model | | Full Model | | Reduced Model | | Full Model | | Reduced Model | |
|  | β | P | β | P | β | P | β | P | β | P | β | P |
| Age at diagnosis | 0.011 | <0.001 | 0.011 | <0.001 | 0.008 | 0.500 | 0.007 | 0.550 | 0.035 | <0.001 | 0.035 | <0.001 |
| Age at diagnosis' | 0.005 | 0.048 | 0.005 | 0.046 | -0.002 | 0.910 | -0.001 | 0.960 | 0.006 | 0.210 | 0.006 | 0.220 |
| Tumor size | 0.023 | <0.001 | 0.023 | <0.001 | 0.007 | 0.430 | 0.006 | 0.520 | 0.008 | 0.002 | 0.006 | 0.018 |
| Tumor size' | -0.018 | <0.001 | -0.018 | <0.001 | 0.000 | 1.000 | 0.002 | 0.870 | -0.014 | <0.001 | -0.013 | 0.002 |
| Examined LNs | -0.048 | <0.001 | -0.049 | <0.001 | -0.028 | 0.210 |  |  | -0.016 | 0.029 |  |  |
| Examined LNs' | 0.043 | <0.001 | 0.044 | <0.001 | 0.034 | 0.220 |  |  | 0.013 | 0.190 |  |  |
| Positive LNs | 0.392 | <0.001 | 0.344 | <0.001 | 0.030 | 0.910 |  |  | -0.057 | 0.610 |  |  |
| Positive LNs' | -0.348 | <0.001 | -0.298 | <0.001 | -0.062 | 0.820 |  |  | 0.054 | 0.650 |  |  |
| Male | 0.169 | <0.001 | 0.165 | <0.001 | 0.128 | 0.260 |  |  | 0.332 | <0.001 | 0.333 | <0.001 |
| Race |  |  |  |  |  |  |  |  |  |  |  |  |
| Asian | -0.157 | <0.001 | -0.158 | <0.001 | -0.242 | 0.330 | -0.254 | 0.310 | -0.346 | <0.001 | -0.348 | <0.001 |
| Black | 0.039 | 0.300 | 0.038 | 0.320 | 0.318 | 0.060 | 0.322 | 0.054 | 0.052 | 0.400 | 0.057 | 0.360 |
| Other/Unknown | -0.488 | 0.007 | -0.483 | 0.007 | -0.556 | 0.580 | -0.549 | 0.590 | -0.012 | 0.960 | -0.012 | 0.960 |
| Lymph node stage |  |  |  |  |  |  |  |  |  |  |  |  |
| N1 | -0.093 | 0.190 |  |  | 0.013 | 0.970 |  |  | -0.048 | 0.730 |  |  |
| N2 | -0.062 | 0.390 |  |  | -0.001 | 1.000 |  |  | -0.003 | 0.980 |  |  |
| N3 | 0.128 | 0.350 |  |  | 0.547 | 0.400 |  |  | 0.601 | 0.038 |  |  |
| Surgery |  |  |  |  |  |  |  |  |  |  |  |  |
| Pneumonectomy | 0.130 | 0.002 | 0.116 | 0.005 | 0.054 | 0.790 |  |  | 0.187 | 0.014 | 0.096 | 0.180 |
| Sub-lobar | 0.145 | <0.001 | 0.141 | <0.001 | 0.109 | 0.530 |  |  | 0.046 | 0.390 | 0.079 | 0.130 |
| Histology subtype |  |  |  |  |  |  |  |  |  |  |  |  |
| ADSC | 0.139 | 0.020 | 0.148 | 0.013 | -0.050 | 0.870 |  |  | 0.178 | 0.062 | 0.186 | 0.050 |
| BAC | -0.110 | 0.006 | -0.106 | 0.008 | -0.086 | 0.680 |  |  | -0.272 | <0.001 | -0.292 | <0.001 |
| LCC | 0.185 | 0.006 | 0.179 | 0.008 | 0.376 | 0.190 |  |  | 0.177 | 0.093 | 0.267 | 0.004 |
| Other | -0.126 | 0.094 | -0.125 | 0.097 | 0.125 | 0.730 |  |  | -0.372 | 0.012 | -0.386 | 0.009 |
| SCC | -0.061 | 0.023 | -0.059 | 0.029 | -0.108 | 0.420 |  |  | 0.342 | <0.001 | 0.366 | <0.001 |
| Unspecified | 0.007 | 0.900 | 0.004 | 0.940 | 0.255 | 0.260 |  |  | 0.219 | 0.008 | 0.255 | 0.001 |
| Extent of tumor |  |  |  |  |  |  |  |  |  |  |  |  |
| Distant | 0.262 | 0.001 | 0.254 | 0.001 | -0.070 | 0.860 |  |  | 0.167 | 0.280 |  |  |
| Regional | 0.200 | <0.001 | 0.196 | <0.001 | 0.012 | 0.920 |  |  | -0.170 | <0.001 |  |  |
| Anatomic sites |  |  |  |  |  |  |  |  |  |  |  |  |
| Middle | 0.060 | 0.230 |  |  | -0.589 | 0.086 |  |  | -0.131 | 0.120 |  |  |
| Lower | 0.116 | <0.001 |  |  | 0.003 | 0.980 |  |  | -0.036 | 0.320 |  |  |
| Bronchus/other | 0.063 | 0.230 |  |  | 0.001 | 1.000 |  |  | -0.153 | 0.120 |  |  |
| Right-sided | 0.046 | 0.041 |  |  | -0.064 | 0.570 |  |  | -0.030 | 0.370 |  |  |
| Clinical stage |  |  |  |  |  |  |  |  |  |  |  |  |
| II | 0.420 | <0.001 | 0.383 | <0.001 | 0.464 | 0.030 | 0.461 | 0.003 | 0.082 | 0.310 |  |  |
| III | 0.530 | <0.001 | 0.516 | <0.001 | 0.558 | 0.007 | 0.550 | <0.001 | 0.032 | 0.670 |  |  |
| IV | 0.957 | <0.001 | 0.953 | <0.001 | 0.820 | <0.001 | 0.848 | <0.001 | -0.236 | 0.023 |  |  |
| Differentiation |  |  |  |  |  |  |  |  |  |  |  |  |
| Moderately | 0.404 | <0.001 | 0.397 | <0.001 | 0.319 | 0.140 | 0.325 | 0.120 | 0.107 | 0.068 |  |  |
| Poorly | 0.573 | <0.001 | 0.563 | <0.001 | 0.439 | 0.051 | 0.496 | 0.018 | 0.106 | 0.087 |  |  |
| Undifferentiated | 0.495 | <0.001 | 0.485 | <0.001 | 0.350 | 0.370 | 0.645 | 0.059 | 0.250 | 0.043 |  |  |
| Chemotherapy | 0.150 | <0.001 | 0.150 | <0.001 | 0.414 | 0.004 | 0.418 | 0.002 | 0.480 | <0.001 | 0.531 | <0.001 |
| Radiotherapy | -0.275 | <0.001 | -0.268 | <0.001 | 0.001 | 0.990 |  |  | 0.038 | 0.540 |  |  |

NOTE. Age at diagnosis', Tumor size', Examined LNs' and Positive LNs' are constructed spline variables (when k = 3).

A model selection technique based on the Bayesian information criteria was used.

eTable 2. Prognostic factors for overall survival by multivariable Cox regression

| Factors | Median OS (95%CI), month | HR (95%CI) | *P*-value |
| --- | --- | --- | --- |
| Diagnostic Age, years |  |  |  |
| <45 | NA | ref |  |
| 45-64 | 96 (91-100) | 1.442 (1.269-1.638) | <0.001 |
| 65-74 | 68 (65-70) | 1.968 (1.731-2.236) | <0.001 |
| ≥75 | 47 (45-48) | 2.765 (2.430-3.147) | <0.001 |
| Gender |  |  |  |
| Female | 85 (82-88) | ref |  |
| Male | 54 (52-55) | 1.321 (1.283-1.361) | <0.001 |
| Ethnicity |  |  |  |
| White | 67 (65-69) | ref |  |
| Asian | 86 (77-98) | 0.783 (0.736-0.833) | <0.001 |
| Black | 68 (63-74) | 1.021 (0.969-1.075) | 0.422 |
| Others/Unknown | 108 (86-NA) | 0.759 (0.609-0.948) | 0.015 |
| Primary tumor location |  |  |  |
| Left-sided | 66 (64-69) | ref |  |
| Right-sided | 70 (68-72) | 1.041 (1.010-1.073) | 0.008 |
| Anatomic sites |  |  |  |
| Upper | 73 (71-75) | ref |  |
| Middle | 70 (63-80) | 0.979 (0.911-1.052) | 0.571 |
| Lower | 63 (61-65) | 1.095 (1.061-1.130) | <0.001 |
| Bronchus/Others | 40 (36-44) | 1.033 (0.962-1.110) | 0.363 |
| Histologic subtype |  |  |  |
| ADC | 73 (71-75) | ref |  |
| SCC | 53 (51-56) | 1.117 (1.078-1.157) | <0.001 |
| BAC | 117 (106-123) | 0.846 (0.799-0.895) | <0.001 |
| ADSC | 45 (39-52) | 1.214 (1.121-1.316) | <0.001 |
| LCC | 42 (35-46) | 1.317 (1.208-1.437) | <0.001 |
| Other | 94 (82-120) | 0.866 (0.774-0.970) | 0.013 |
| Unspecified | 47 (43-54) | 1.077 (1.005-1.154) | 0.034 |
| Differentiation |  |  |  |
| Well | 123 (118-NA) | ref |  |
| Moderately | 72 (70-74) | 1.395 (1.316-1.478) | <0.001 |
| Poorly | 50 (48-52) | 1.606 (1.513-1.705) | <0.001 |
| Undifferentiated | 44 (38-54) | 1.530 (1.372-1.707) | <0.001 |
| Clinical stage |  |  |  |
| I | 98 (96-100) | ref |  |
| II | 42 (40-44) | 1.431 (1.345-1.522) | <0.001 |
| III | 35 (33-36) | 1.505 (1.415-1.600) | <0.001 |
| IV | 24 (22-26) | 2.430 (2.277-2.592) | <0.001 |
| Tumor size, cm |  |  |  |
| ≤1.0 | NA | ref |  |
| 1.1 to 3.0 | 86 (84-90) | 1.296 (1.181-1.422) | <0.001 |
| 3.1 to 5.0 | 52 (50-54) | 1.652 (1.502-1.817) | <0.001 |
| 5.1 to 7.0 | 37 (34-40) | 1.887 (1.706-2.088) | <0.001 |
| >7.1 | 24 (22-26) | 2.429 (2.185-2.700) | <0.001 |
| Tumor extent |  |  |  |
| Local | 82 (80-84) | ref |  |
| Regional | 46 (45-48) | 1.190 (1.153-1.229) | <0.001 |
| Distant | 17 (15-21) | 1.536 (1.381-1.709) | <0.001 |
| Lymph node stage |  |  |  |
| N0 | 89 (87-91) | ref |  |
| N1 | 38 (37-40) | 0.840 (0.746-0.945) | 0.004 |
| N2 | 30 (29-31) | 0.979 (0.871-1.100) | 0.724 |
| N3 | 18 (16-22) | 1.488 (1.209-1.831) | <0.001 |
| Examined lymph node |  |  |  |
| <5 | 62 (60-65) | ref |  |
| 5 to 9 | 72 (70-75) | 0.834 (0.803-0.866) | <0.001 |
| 10 to 14 | 72 (69-77) | 0.756 (0.723-0.790) | <0.001 |
| 15 to 20 | 71 (67-77) | 0.698 (0.660-0.739) | <0.001 |
| ≥20 | 63 (59-69) | 0.668 (0.632-0.707) | <0.001 |
| Positive lymph node |  |  |  |
| 0 | 88 (86-91) | ref |  |
| 1 | 39 (37-42) | 1.507 (1.347-1.685) | <0.001 |
| 2 | 35 (32-38) | 1.633 (1.454-1.834) | <0.001 |
| 3 | 30 (28-34) | 1.814 (1.604-2.051) | <0.001 |
| ≥4 | 26 (25-28) | 2.170 (1.937-2.432) | <0.001 |
| Type of surgery |  |  |  |
| Lobectomy | 74 (72-76) | ref |  |
| Pneumonectomy | 30 (28-33) | 1.148 (1.086-1.215) | <0.001 |
| Sub-lobar | 58 (55-61) | 1.176 (1.121-1.235) | <0.001 |
| Chemotherapy |  |  |  |
| None | 77 (75-79) | ref |  |
| Yes | 50 (48-52) | 0.705 (0.679-0.732) | <0.001 |
| Radiotherapy |  |  |  |
| None | 78 (75-79) | ref |  |
| Yes | 31 (30-32) | 1.288 (1.233-1.344) | <0.001 |

**
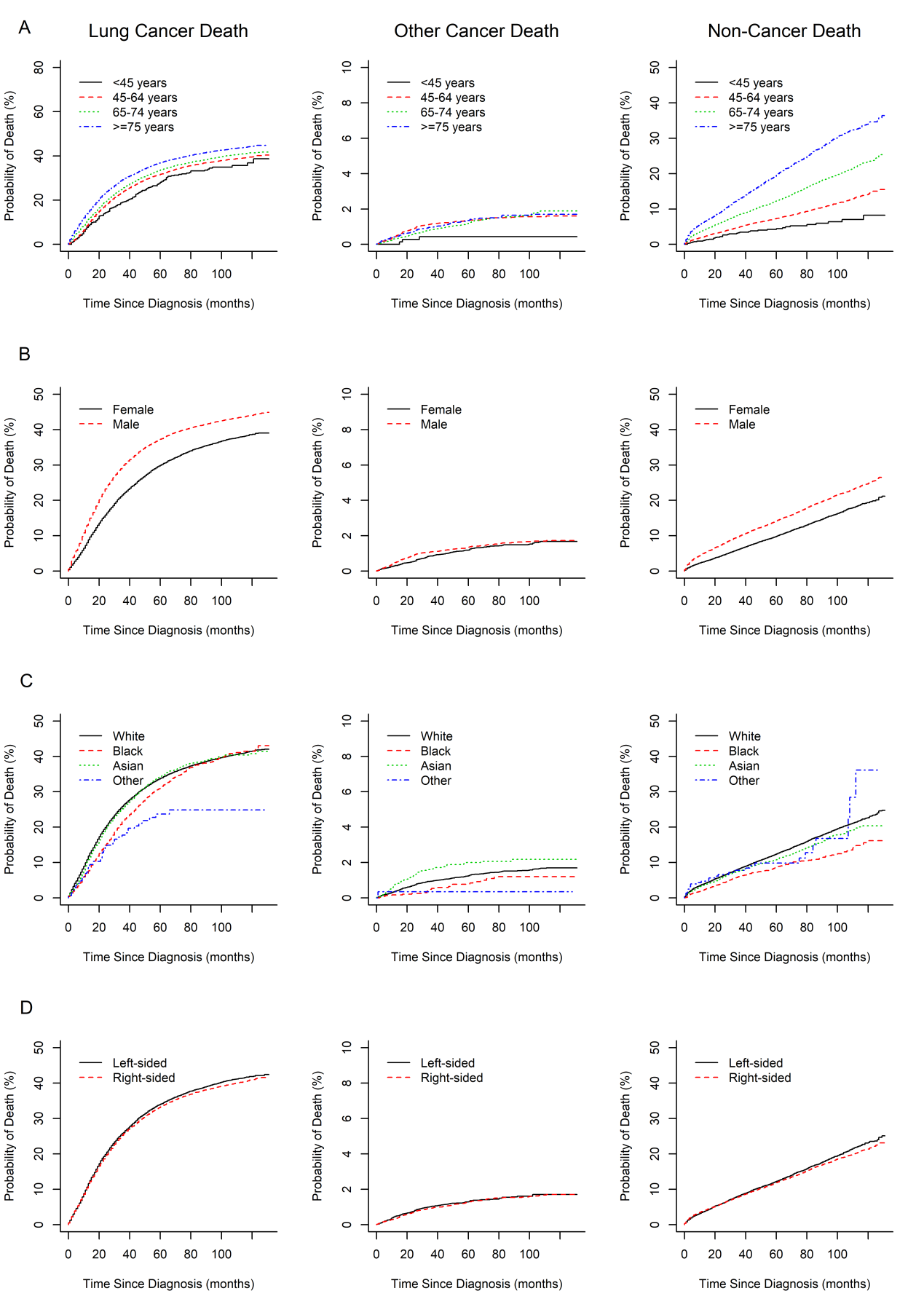
**

**eFigure 1.** Lung cancer related, other cancer related and non-cancer related death rates by (A) age, (B) gender, (C) race and (D) primary tumor location.

**
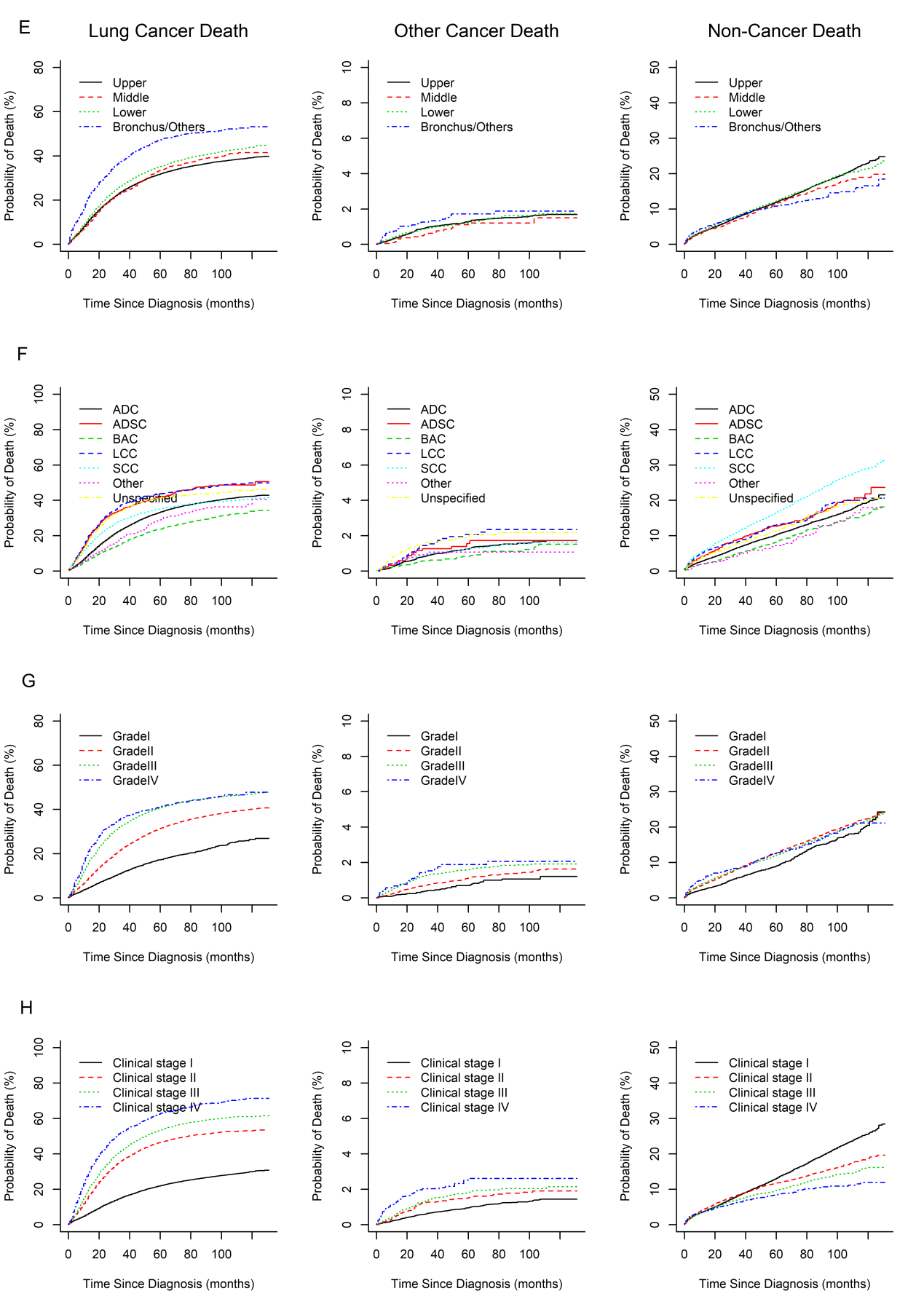
**

**eFigure 2.** Lung cancer related, other cancer related and non-cancer related death rates by (E) Anatomic sites, (F) histology subtype, (G) differentiation and (H) clinical stage.

**
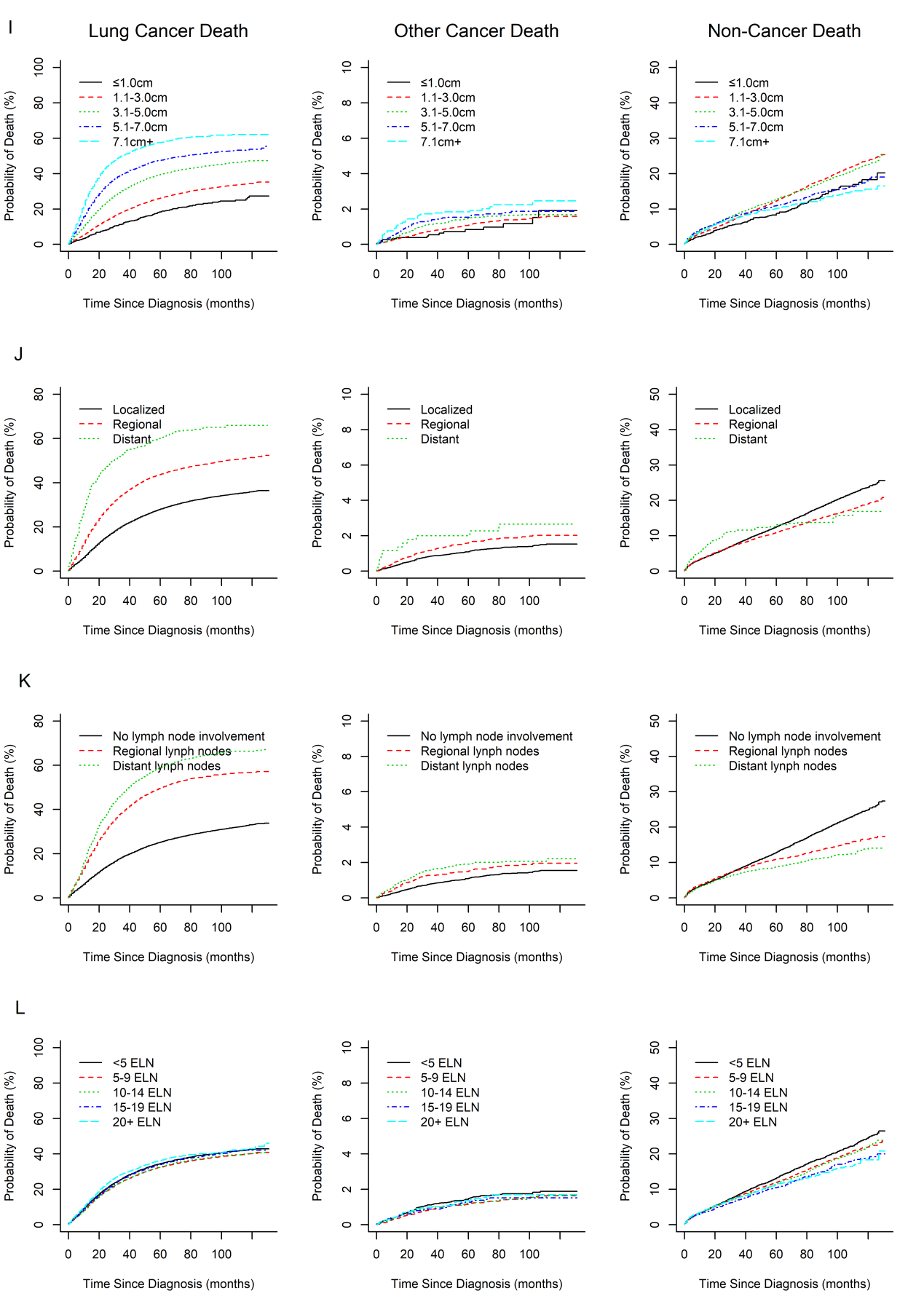
**

**eFigure 3.** Lung cancer related, other cancer related and non-cancer related death rates by (I) tumor size, (J) tumor extent, (K) lymph node involvement and (L) examined lymph nodes.

**
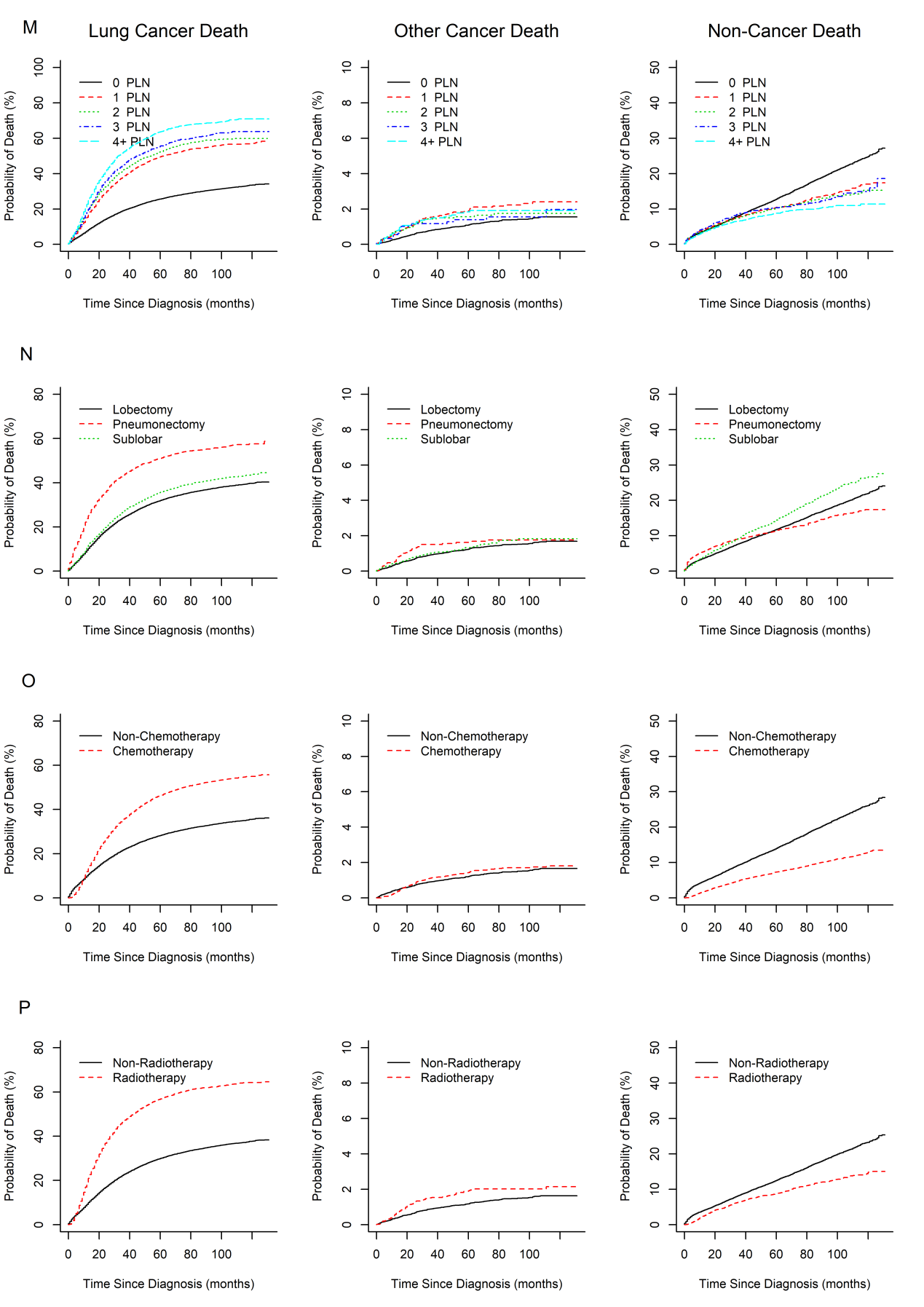
**

**eFigure 4.** Lung cancer related, other cancer related and non-cancer related death rates by (M) positive lymph nodes, (N) surgery, (O) chemotherapy and (P) radiotherapy.

**
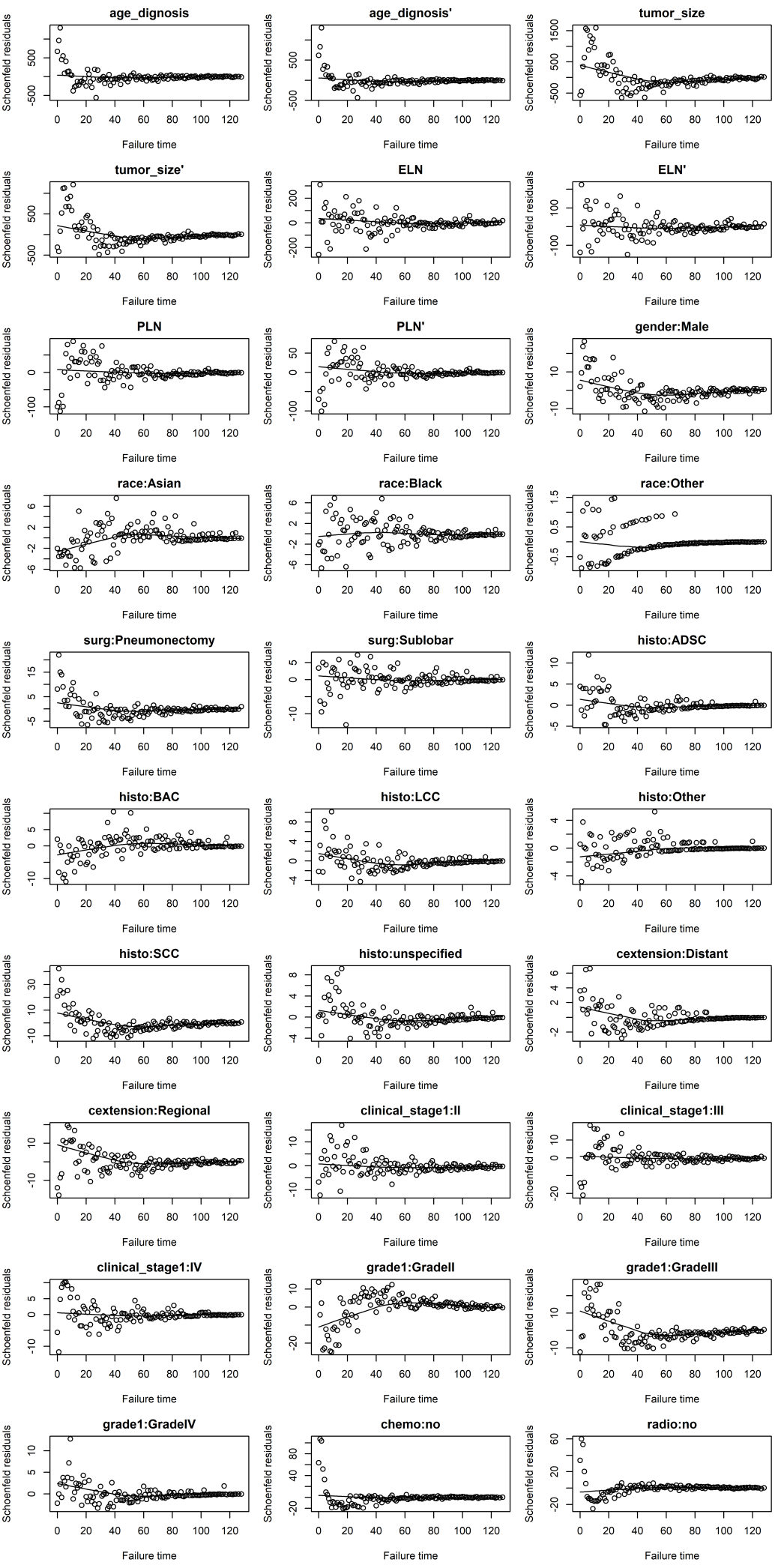
**

**eFigure 5.** Schoenfeld−type residuals of a proportional subdistribution hazard model for lung cancer related deaths.
